# TRACK Implementation: a Bangladesh Scenario

**DOI:** 10.5195/cajgh.2020.416

**Published:** 2020-05-26

**Authors:** Abdul Kader Mohiuddin

**Affiliations:** 1 Dr. M. Nasirullah Memorial Trust, Tejgaon, Dhaka, Bangladesh

**Keywords:** Blood sugar Screening, Compliance, Overweight, Lifestyle, Regular health checkup, Ramadan fasting, Climate issue of diabetes

## Abstract

With the increasing burden of non-communicable diseases in low-income and middle-income countries (LMICs), biological risk factors, such as hyperglycemia, are a major public health concern in Bangladesh. Optimization of diabetes management by positive lifestyle changes is urgently required for prevention of comorbidities and complications, which in turn will reduce the cost. Diabetes had 2 times more days of inpatient treatment, 1.3 times more outpatient visits, and nearly 10 times more medications than non-diabetes patients, as reported by British Medical Journal. And surprisingly, 80% of people with this so called “Rich Man's Disease” live in low- and middle-income countries. According to a recent study of American Medical Association, China and India collectively are home of nearly 110 million diabetic patients. The prevalence of diabetes in this region is projected to increase by 71% by 2035. Bangladesh was ranked as the 8th highest diabetic populous country in the time period of 2010-2011. In Bangladesh, the estimated prevalence of diabetes among adults was 9.7% in 2011 and the number is projected to be 13.7 million by 2045. The cost of diabetes care is considerably high in Bangladesh, and it is primarily driven by the medicine and hospitalization costs. According to Bangladesh Bureau of Statistics, in 2017 the annual average cost per T2DM was $864.7, which is 52% of per capita GDP of Bangladesh and 9.8 times higher than the general health care cost. Medicine is the highest source of direct cost (around 85%) for patients without hospitalization. The private and public financing of diabetes treatment will be severely constrained in near future, representing a health threat for the Bangladeshi population.

Bangladesh was ranked as the 8th highest diabetic populous country in the time period of 20102011 [1]. In Bangladesh, the estimated prevalence of diabetes among adults was 9.7% in 2011 and the number is projected to be 13.7 million by 2045. The cost of diabetes care is considerably high in Bangladesh, and it is primarily driven by the medicine and hospitalization costs. According to Bangladesh Bureau of Statistics, in 2017 the annual average cost per T2DM was $864.7, which is 52% of per capita GDP of Bangladesh and 9.8 times higher than the general health care cost [2].

In Bangladesh, specifically, the IDF projects the prevalence of diabetes will increase to more than 50% in the next 15 years [3,4]. About 129,000 deaths were attributed to diabetes in Bangladesh in 2015, as reported by leading research organization ICDDR, B [5]. According to the WHO-Diabetes country profile of Bangladesh in 2016, the physical inactivity was prevailing among 25.1% of population [6]. Around 85% population of age group 25-65 never checks for diabetes [7]. A recent study by British Medical Journal says, 1 in 10 Bangladeshi adults aged ≥18 years have hyperglycemia (among urban residents) [8]. Even in rural Bangladeshi community, undiagnosed diabetes was high, 7.2% found in a 2016 and 10% in 2019 [9,10]. Roughly 20%-30% of adults in rural areas of Bangladesh have abnormal fasting glucose or impaired glucose tolerance, with the prevalence of diabetes (mostly type 2 diabetes) expected to reach 24%-34% by 2030 [10–12]. And IDF says, there are 7.1 million people with undetected diabetes in Bangladesh and this number will be double by 2025 [13]. Prevalence of dyslipidemia was over 70% to both male and female subjects, which indicates the urgency of lifestyle intervention strategies to prevent and manage this important health problem and risk factor [15]. Among 8400 stroke patients from different hospitals in Bangladesh over a period of sixteen years, diabetic patients were nearly 25% [16].

ICDDR, B, estimated 150 food items in the country. More than 50% of the food samples they tested were adulterated reported by the Institute of Public Health (IPH) [17]. Undoubtedly human health is now under the domination of formalin, in Bangladesh about 400 tons of formalin is being imported which are goes to human stomach, creates deadly mistreats on long term exposure [18]. Several studies highlighted formaldehyde-induced neurodegeneration, diabetes risk and diabetes-associated cognitive impairments [19–21]. Even more unfortunate is the fact that nefarious practice of food adulteration increases exponentially during the month of Ramadan in Bangladesh [22].

**Figure 1.**
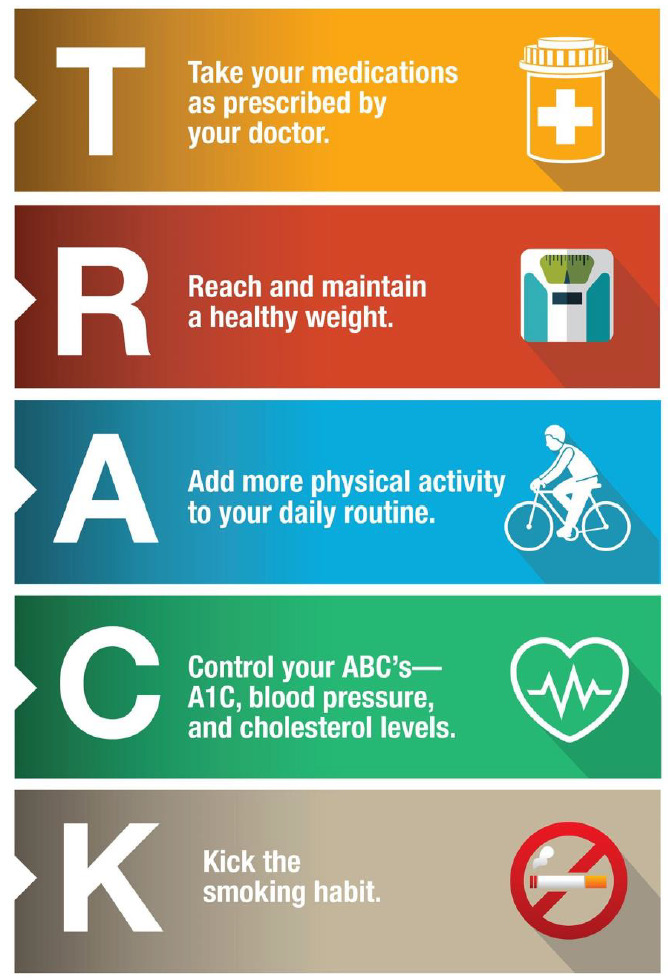
TRACK, a program of National Institute of Health (NIH), England to memorize the factors that can contribute to health while living with diabetes [14].

**Table 1.** Summary of Diabetic Risk Factors in Bangladesh

Risk Factors	Prevalence
Physical inactivity (overall)	25.1%
Physical inactivity (among adults)	35% to 38%
Young adults among capital who unmet recommended physical activity	80%
Adults who never checks diabetes	85%
Undiagnosed diabetes among rural population	7.2%
Adults with hyperglycemia	10%
Abnormal fasting glucose among rural population	20%-30%
People over the age of 35 having diabetes under control	12%
People over 35 had abnormal fasting glucose	25%
Stroke among diabetic patients	25%
Non-compliance with medication	87%
Prevalence of dyslipidemia	More than 70%
Obesity among young adults	22% to 27%
Obesity among school going children	40%
Mothers unaware of consequences of childhood obesity	70%
Obesity among urban women	34%
Obesity among married women	30%
Obesity increase among women in 15 years study	17.5%
Higher prevalence of diabetes among males	7.4%
Overall consumption of fast food consumption among youth and children	Around 54%
Prevalence of self-reported depression	47%
Smokers (male)	37%
GDM	15%
Adulterated food in daily consumption	50%
Child marriage	30%
Undernourished women	33%
Underweight among children aged less than five years	40%
Low health literacy (among urban people)	60%

A Netherlands based study in CNN Health says, “a 1-degree Celsius rise in environmental temperature could account for more than 100,000 new diabetes cases per year in the USA alone” [23]. A similar study says Bangladesh will exceed 35-degree Celsius before the end of the century [24]. Consuming arsenic contaminated food grains could be another reason of high diabetes prevalence [25]. In sex-stratified analyses with 641 subjects from rural Bangladesh, a study reported arsenic exposure (50.01-150 μg/L) showed a clearer pattern of dose-dependent risk for hyperglycemia in females than males [26]. Again, 15% of expecting women are diagnosed with gestational diabetes among these 60% contribute to permanent diabetes within 10 years, says Dr Samsad Jahan (professor of Obstetrics and Gynecology, BIRDEM) [27].

According to a 2018 BBC record, insulin availability found supplies were low in six countries - Bangladesh, Brazil, Malawi, Nepal, Pakistan and Sri Lanka [28,29]. Also, huge gap between the number of diabetic patients and doctors are well-known [30]. The Diabetic Association of Bangladesh (DAB) record shows, except Dhaka and Chittagong, there are no tertiary facilities in Bangladesh to preventing blindness due to diabetic retinopathy. Children with diabetes are still managed by adult physicians or occasionally by adult diabetologists, except in institutions like BIRDEM, and Dhaka Shishu Hospital [31].

Around 22% to 27% Bangladeshi youth were recorded as obese with different stages of obesity [32]. Another study says nearly 40% Bangladeshi youth, taking fast foods were recognized as overweight where 32% were noted as obese with different phases of obesity and overall prevalence of fast food consumption was about 53.8% [33]. In a newspaper interview, Professor AK Azad Khan, President, Diabetic Association of Bangladesh said 40% school going children of Dhaka city were either obese or overweight [34]. “Children with type 2 diabetes is rising “alarmingly” in Bangladesh. A 300% raise in the last five years”, according to the Changing Diabetes in Children Program of the BIRDEM hospital [35]. A community level study shows 35% of mothers perceived that childhood overweight/obesity could be a health problem and nearly 70% were not aware of any health consequences of childhood obesity [36]. Another study shows 97.4% students consume fast food contain Monosodium Glutamate which causes obesity and other body discomforts [37]. In a similar study among students of 4 private universities of Dhaka, 98% of the students were well informed about the negative effects associated with excessive fast food consumption, they were still profoundly addicted to it [38]. Prevalence rates of overweight and obesity are higher in urban peoples compared to rural peoples living in Bangladesh [39].

According to the WHO-Diabetes country profile of Bangladesh in 2016, the physical inactivity was prevailing more than 25% of population. Bangladeshi women more at health risk than men due to inactivity. Two big reasons to diabetes among Bangladeshi people are carbohydrate-dependent food pattern and sedentary lifestyle [40–42]. Evidence shows that prevalence of physical inactivity 35% to 38% in Bangladeshi adults aged 25 years and older [43].

Despite the high levels of diabetes and intermediate hyperglycemia, awareness and control of the condition is low [44]. In a cross-sectional study in urban population of Bangladesh, more than 60% of the diabetic patients had inadequate functional health literacy of them and nearly 90% had inadequate glycemic control (HbA1c>8%) [45]. Also, another study says that diabetes-related health literacy in rural Bangladesh is a major factor associated with diabetic retinopathy (DR) screening [46].

The IDF atlas estimated the incidence of type 1 diabetes in Bangladesh as 4.2 new cases of T1DM/100,000 children (0-14 years)/year, in 2013 [31]. The social challenges faced by T1DM children are numerous. Many of them are poor, with little access to education. They are often considered a burden on the family, especially girls; they have little prospect of getting married or being employed. According to UNICEF, Bangladesh has the fourth highest prevalence rate of child marriage in the world, and the second highest number of absolute child brides - 4.5 million. Around 30% of girls in Bangladesh married before the age of 15 and nearly 80% got married before the age of 18 [47–49]. The prevalence of nutritional deficiency was relatively higher among rural, illiterate and early married women and among those with a low standard of living. Child marriage, low-birthweight, mother nutrition and diabetes closely related to each other [50].

Recently, Telenor Health and DAB have launched the first-ever diabetes management service, Dia360, to help people with diabetes manage their blood sugar levels and reduce risks of complications. People can enroll in three DAB centers in Dhaka—Bangladesh Institute of health and Sciences, Bangladesh Institute of Research and Rehabilitation in Diabetes, Endocrine and Metabolic Disorders (BIRDEM) General Hospital, and the National Health Network Hospital. It has more than 400,000 diabetics registered at its tertiary center, BIRDEM in Dhaka. However, the most important thing is patient education, that the modern world is giving the highest priorities. Rich or poor, privileged or unprivileged, all segment of population should be brought under the arena of compliance through patient education, at least by health campaign. Both government, profit taking NGOs and pharmaceutical companies should take initiatives in this regard.

## Abbreviations

LMICs: Low- And Middle-Income Countries
IDF: International Diabetes Federation
NEHEP: National Eye Health Education Program
BDHS: Bangladesh Demographic and Health Survey
BIRDEM: Bangladesh Institute of Research and Rehabilitation in Diabetes, Endocrine and Metabolic Disorders
IPH: Institute of Public Health

## References

[1] Bhowmik B, Binte Munir S, Ara Hossain I, Siddiquee T, Diep LM, Mahmood S, Mahtab H, Khan AK, Hussain A Prevalence of type 2 diabetes and impaired glucose regulation with associated cardiometabolic risk factors and depression in an urbanizing rural community in bangladesh: a population-based cross-sectional study. Diabetes Metab J. 2012 12;36(6):422–32. doi: 10.4093/dmj.2012.36.6.422. 23275936 PMC3530713

[2] Afroz A, Alam K, Ali L, et al. Type 2 diabetes mellitus in Bangladesh: a prevalence based cost-of-illness study. BMC Health Serv Res. 2019;19(1):601 Published 2019 8 27. doi:10.1186/s12913-019-4440-3 31455307 PMC6712789

[3] Islam JY, Zaman MM, Bhuiyan MR, Haq SA, Ahmed S, Al-Qadir AZ Prevalence and determinants of hyperglycaemia among adults in Bangladesh: results from a population-based national survey. BMJ Open. 2019;9(7):e029674 Published 2019 7 24. doi:10.1136/bmjopen-2019-029674 PMC6661587 31345979

[4] Rawal LB, Biswas T, Khandker NN, et al. Non-communicable disease (NCD) risk factors and diabetes among adults living in slum areas of Dhaka, Bangladesh. PLoS One. 2017;12(10):e0184967 Published 2017 10 3. doi:10.1371/journal.pone.0184967 28972972 PMC5626026

[5] Mohiuddin AK Diabetes Fact: Bangladesh Perspective. International Journal of Diabetes Research. 2019;2(1):14–20. doi:10.17554/j.issn.2414-2409.2019.02.12.

[6] Salahuddin T Obesity is increasing among the younger generation in Bangladesh. The Daily Star, Bangladesh, 9 23, 2018

[7] Star Online Report. 80 lakh Bangladeshis suffering from diabetes: State minister. The Daily Star, 4 06, 2016.

[8] Asaduzzaman M, Chowdhury S, Shahed JH, et al. Prevalence of Type 2 Diabetes Mellitus Among Urban Bihari Communities in Dhaka, Bangladesh: A Cross-sectional Study in a Minor Ethnic Group. Cureus. 2018;10(1):e2116 Published 2018 1 26. doi:10.7759/cureus.2116 29593946 PMC5871323

[9] Islam FM, Chakrabarti R, Islam MT, Wahab M, Lamoureux E, Finger RP, Shaw JE Prediabetes, diagnosed and undiagnosed diabetes, their risk factors and association with knowledge of diabetes in rural Bangladesh: The Bangladesh Population-based Diabetes and Eye Study. J Diabetes. 2016 3;8(2):260–8. doi: 10.1111/1753-0407.12294. 25851830

[10] Fottrell E, Ahmed N, Morrison J, et al. Community groups or mobile phone messaging to prevent and control type 2 diabetes and intermediate hyperglycaemia in Bangladesh (DMagic): a cluster-randomised controlled trial. Lancet Diabetes Endocrinol. 2019;7(3):200–212. doi:10.1016/S2213-8587(19)30001-4 30733182 PMC6381080

[11] Fottrell E, Ahmed N, Shaha SK, et al. Distribution of diabetes, hypertension and non-communicable disease risk factors among adults in rural Bangladesh: a cross-sectional survey. BMJ Glob Health. 2018;3(6):e000787 Published 2018 11 12. doi:10.1136/bmjgh-2018-000787 PMC6242007 30498584

[12] Hasan MM, Tasnim F, Tariqujjaman M, Ahmed S Socioeconomic Inequalities of Undiagnosed Diabetes in a Resource-Poor Setting: Insights from the Cross-Sectional Bangladesh Demographic and Health Survey 2011. Int J Environ Res Public Health. 2019;16(1):115 Published 2019 1 3. doi:10.3390/ijerph16010115 PMC6338882 30609855

[13] Shariful Islam SM, Lechner A, Ferrari U, Laxy M, Seissler J, Brown J, Niessen LW, Holle R Healthcare use and expenditure for diabetes in Bangladesh. BMJ Glob Health. 2017 1 3;2(1):e000033. doi: 10.1136/bmjgh-2016-000033. PMC5321382 28588991

[14] Wisconsin Council of the Blind & Visually Impaired. Stay on TRACK of Your Diabetes. wp-content, 5 28, 2019 Available In: https://wcblind.org/wp-content/uploads/2019/06/NYCU-June-2019-pdf.pdf

[15] Das H, Banik S Prevalence of dyslipidemia among the diabetic patients in southern Bangladesh: A cross-sectional study. Diabetes Metab Syndr. 2019 Jan-Feb;13(1):252–257. doi: 10.1016/j.dsx.2018.09.006. 30641707

[16] Mohammad QD, Habib M, Mondal BA, Chowdhury RN, Hasan MH, Hoque MA, Rahman KM, Khan SU, Chowdhury AH, Haque B Stroke in Bangladeshi patients and risk factor. Mymensingh Med J. 2014 7;23(3):520–9. 25178605

[17] Mohiuddin AK The Mysterious Domination of Food Contaminants and Adulterants in Bangladesh. Journal of Environmental Science and Public Health. 2018;03(01):34–56. doi:10.26502/jesph.96120046.

[18] Tulpule K, Dringen R Formaldehyde in brain: an overlooked player in neurodegeneration? J Neurochem. 2013 10;127(1):7–21. doi: 10.1111/jnc.12356. 23800365

[19] Tan T, Zhang Y, Luo W, Lv J, Han C, Hamlin JNR, Luo H, Li H, Wan Y, Yang X, Song W, Tong Z Formaldehyde induces diabetes-associated cognitive impairments. FASEB J. 2018 7;32(7):3669–3679. doi: 10.1096/fj.201701239R. 29401634

[20] Hipkiss AR Depression, Diabetes and Dementia: Formaldehyde May Be a Common Causal Agent; Could Carnosine, a Pluripotent Peptide, Be Protective?. Aging Dis. 2017;8(2):128–130. Published 2017 4 1. doi:10.14336/AD.2017.0120 28400979 PMC5362172

[21] Ai L, Tan T, Tang Y, et al. Endogenous formaldehyde is a memory-related molecule in mice and humans. Commun Biol. 2019;2:446 Published 2019 11 29. doi:10.1038/s42003-019-0694-x 31815201 PMC6884489

[22] Mohiuddin AK Chemical Contaminants and Pollutants in the Measurable Life of Dhaka City. European Journal of Sustainable Development Research. 2019;3(2), em0083 10.29333/ejosdr/5727

[23] Azad A How climate change will affect your health. CNN health, 10 12, 2018.

[24] Tribune Desk. Temperature in Bangladesh to raise to deadly heights by end of century. Dhaka Tribune, Bangladesh, 8 03, 2017.

[25] Mohiuddin AK Domination of Pollutant Residues among Food Products of South-East Asian Countries. Global Journal of Nutrition & Food Science. 2019;2(3):1–4. doi:10.33552/gjnfs.2019.02.000536.

[26] Paul SK, Islam MS, Hasibuzzaman MM, Hossain F, Anjum A, Saud ZA, Haque MM, Sultana P, Haque A, Andric KB, Rahman A, Karim MR, Siddique AE, Karim Y, Rahman M, Miyataka H, Xin L, Himeno S, Hossain K Higher risk of hyperglycemia with greater susceptibility in females in chronic arsenic-exposed individuals in Bangladesh. Sci Total Environ. 2019 6 10;668:1004–1012. doi: 10.1016/j.scitotenv.2019.03.029. 31018442 PMC6560360

[27] Chaity AJ 15% pregnant women diagnosed with diabetes. DhakaTribune, 11 14th, 2017.

[28] Biswas S Is the world heading for an insulin shortage? BBC News, ASIA/India, 30 11 2018.

[29] Beran D, Ewen M, Laing R Constraints and challenges in access to insulin: a global perspective. The Lancet Diabetes & Endocrinology. 2016;4(3):275–285. doi:10.1016/s2213-8587(15)00521-5. 26857998

[30] Rahman MS, Akter S, Abe SK, et al. Awareness, treatment, and control of diabetes in Bangladesh: a nationwide population-based study. PLoS One. 2015;10(2):e0118365 Published 2015 2 18. doi:10.1371/journal.pone.0118365 25692767 PMC4334658

[31] Azad K Type 1 diabetes: The Bangladesh perspective. Indian Journal of Endocrinology and Metabolism. 2015;19(7):9–11. doi:10.4103/2230-8210.155344. PMC4413401 25941662

[32] Al Muktadir MH, Islam MA, Amin MN, Ghosh S, Siddiqui SA, Debnath D, Islam MM, Ahmed T, Sultana F Nutrition transition - Pattern IV: Leads Bangladeshi youth to the increasing prevalence of overweight and obesity. Diabetes Metab Syndr. 2019 May-Jun;13(3):1943–1947. doi: 10.1016/j.dsx.2019.04.034. 31235119

[33] Goon S, Bipasha MS, Islam MS Fast Food Consumption and Obesity Risk among University Students of Bangladesh. European Journal of Preventive Medicine. 2014;2(6):99–104. doi: 10.11648/j.ejpm.20140206.14

[34] The Daily Star. “Access to insulin is a human right”. World Diabetes Day 2018, 11 14, 2018.

[35] Hasib NI Children getting type 2 diabetes alarmingly in Bangladesh. bdnews 24.com, Bangladesh, 06 4, 2016.

[36] Hossain MS, Siddiqee MH, Ferdous S, Faruki M, Jahan R, Shahik SM, Raheem E, Okely AD Is Childhood Overweight/Obesity Perceived as a Health Problem by Mothers of Preschool Aged Children in Bangladesh? A Community Level Cross-Sectional Study. Int J Environ Res Public Health. 2019 1 12;16(2). pii: E202. doi: 10.3390/ijerph16020202. 30642056 PMC6352241

[37] Chaity AJ Obesity blamed for alarming rise in childhood diabetes. Dhaka Tribune, Bangladesh, 11 13, 2017.

[38] Bipasha MS, Goon S Fast food preferences and food habits among students of private universities in Bangladesh. South East Asia Journal of Public Health . 2014;3(1):61–64. 10.3329/seajph.v3i1.17713

[39] Hoque ME, Long KZ, Niessen LW, Al Mamun A Rapid shift toward overweight from double burden of underweight and overweight among Bangladeshi women: a systematic review and pooled analysis. Nutr Rev. 2015 7;73(7):438–47. doi: 10.1093/nutrit/nuv003. 26081454

[40] Guthold R, Stevens GA, Riley LM, Bull FC Worldwide trends in insufficient physical activity from 2001 to 2016: a pooled analysis of 358 population–based surveys with 19 million participants. Lancet Glob Health. 2018 10;6(10):e1077–e1086. doi: 10.1016/S2214-109X(18)30357-7. 30193830

[41] Mahbub I Why Is Diabetes on The Rise in Bangladesh? Future Startup 10 25, 2016.

[42] Tareq S Obesity is increasing among the younger generation in Bangladesh. The Daily Star, Bangladesh, 9 23, 2018.

[43] Vancampfort D, Firth J, Schuch F, Rosenbaum S, De Hert M, Mugisha J, Probst M, Stubbs B Physical activity and sedentary behavior in people with bipolar disorder: A systematic review and meta-analysis. J Affect Disord. 2016 9 1;201:145–52. doi: 10.1016/j.jad.2016.05.020. 27235817

[44] WHO/Country office for Bangladesh. Double trouble: diabetes and depression. Available In: http://www.searo.who.int/bangladesh/depressiondoubletrouble/en/

[45] Mehzabin R, Hossain KJ, Moniruzzaman M, Sayeed SKJB Association of Functional Health Literacy with Glycemic Control: A Cross Sectional Study in Urban Population of Bangladesh. Journal of Medicine. 2019;20(1):19–24. doi:10.3329/jom.v20i1.38816.

[46] Islam FMA, Kawasaki R, Finger RP Factors associated with participation in a diabetic retinopathy screening program in a rural district in Bangladesh. Diabetes Res Clin Pract. 2018 10;144:111–117. doi: 10.1016/j.diabres.2018.08.012. 30142363

[47] MARRY BEFORE YOUR HOUSE IS SWEPT AWAY Child Marriage in Bangladesh. Human Rights Documents Online. doi:10.1163/2210-7975hrd-2156-2015030.

[48] Kamal SM, Hassan CH, Alam GM, Ying Y Child marriage in Bangladesh: trends and determinants. J Biosoc Sci. 2015 1;47(1):120–39. doi: 10.1017/S0021932013000746. 24480489

[49] Hossain MG, Mahumud RA, Saw A PREVALENCE OF CHILD MARRIAGE AMONG BANGLADESHI WOMEN AND TREND OF CHANGE OVER TIME. J Biosoc Sci. 2016 8;48(4):530–8. doi: 10.1017/S0021932015000279. 26286142

[50] Zahangir MS, Hasan MM, Richardson A, Tabassum S Malnutrition and non-communicable diseases among Bangladeshi women: an urban-rural comparison. Nutr Diabetes. 2017 3 20;7(3):e250. doi: 10.1038/nutd.2017.2. 28319102 PMC5380895

